# Additional Effects of Nutritional Antioxidant Supplementation on Peripheral Muscle during Pulmonary Rehabilitation in COPD Patients: A Randomized Controlled Trial

**DOI:** 10.1155/2019/5496346

**Published:** 2019-04-17

**Authors:** Fares Gouzi, Jonathan Maury, Nelly Héraud, Nicolas Molinari, Héléna Bertet, Bronia Ayoub, Marine Blaquière, François Bughin, Philippe De Rigal, Magali Poulain, Joël Pincemail, Jean-Paul Cristol, Dalila Laoudj-Chenivesse, Jacques Mercier, Christian Préfaut, Pascal Pomiès, Maurice Hayot

**Affiliations:** ^1^PhyMedExp, Montpellier University, INSERM U1046, CNRS UMR 9214, Montpellier University Hospital, France; ^2^PhyMedExp, Montpellier University, INSERM U1046, CNRS UMR 9214, France; ^3^Pulmonary Rehabilitation Centers Les Cliniques du Souffle®, 5 Santé/Fontalvie Corporation, 66350 Toulouges, France; ^4^IMAG, CNRS, Montpellier University, Montpellier University Hospital, Montpellier, France; ^5^Department of Cardiovascular Surgery and CREDEC, University of Liège, Sart Tilman University Hospital, 4000 Liège, Belgium; ^6^Montpellier University, France

## Abstract

**Background:**

Skeletal muscle dysfunction in patients with chronic obstructive pulmonary disease (COPD) is not fully reversed by exercise training. Antioxidants are critical for muscle homeostasis and adaptation to training. However, COPD patients experience antioxidant deficits that worsen after training and might impact their muscle response to training. Nutritional antioxidant supplementation in combination with pulmonary rehabilitation (PR) would further improve muscle function, oxidative stress, and PR outcomes in COPD patients.

**Methods:**

Sixty-four COPD patients admitted to inpatient PR were randomized to receive 28 days of oral antioxidant supplementation targeting the previously observed deficits (PR antioxidant group; *α*-tocopherol: 30 mg/day, ascorbate: 180 mg/day, zinc gluconate: 15 mg/day, selenomethionine: 50 *μ*g/day) or placebo (PR placebo group). PR consisted of 24 sessions of moderate-intensity exercise training. Changes in muscle endurance (primary outcome), oxidative stress, and PR outcomes were assessed.

**Results:**

Eighty-one percent of the patients (FEV_1_ = 58.9 ± 20.0%pred) showed at least one nutritional antioxidant deficit. Training improved muscle endurance in the PR placebo group (+37.4 ± 45.1%, *p* < 0.001), without additional increase in the PR antioxidant group (-6.6 ± 11.3%; *p* = 0.56). Nevertheless, supplementation increased the *α*-tocopherol/*γ*-tocopherol ratio and selenium (+58 ± 20%, *p* < 0.001, and +16 ± 5%, *p* < 0.01, respectively), muscle strength (+11 ± 3%, *p* < 0.001), and serum total proteins (+7 ± 2%, *p* < 0.001), and it tended to increase the type I fiber proportion (+32 ± 17%, *p* = 0.07). The prevalence of muscle weakness decreased in the PR antioxidant group only, from 30.0 to 10.7% (*p* < 0.05).

**Conclusions:**

While the primary outcome was not significantly improved, COPD patients demonstrate significant improvements of secondary outcomes (muscle strength and other training-refractory outcomes), suggesting a potential “add-on” effect of the nutritional antioxidant supplementation (vitamins C and E, zinc, and selenium) during PR. This trial is registered with NCT01942889.

## 1. Introduction

Chronic obstructive pulmonary disease (COPD) is systematically associated with comorbidities [1]. Peripheral muscle dysfunction, characterized by reduced endurance, atrophy, and weakness, is a common comorbidity that negatively impacts prognosis [2]. Muscle atrophy and weakness are therefore targets of exercise training interventions in pulmonary rehabilitation (PR) [3]. However, the available evidence suggests that the muscle response to training is blunted in COPD patients, with either no or subphysiological responses regarding muscle strength, fiber size, and oxidative fibers [4, 5]. Although still debated [2], oxidative stress and antioxidant deficits could impair the muscle response to training in COPD.

Oxidative stress is indeed a deleterious factor leading to muscle dysfunction and atrophy in COPD [2]. It results from an imbalance between excessive reactive oxygen species (ROS) production and a deficit in antioxidant defenses. *In vitro* experiments in COPD atrophic myotubes [6] match the clinical resting and exercise evidence [7]. Specifically, antioxidants play a key role as they improve muscle endurance [8] and atrophy in COPD [6].

Antioxidants also physiologically prevent muscle dysfunction and atrophy by buffering ROS [7], lowering systemic inflammation [9], and modulating cell functions [10]. Alpha-tocopherol, zinc, and selenium can protect the muscle against oxidative stress and inflammation *in vitro* and *in vivo* [11, 12] and are critical to muscle homeostasis [13]. The combination of antioxidant deficiencies impairs the muscle redox state [13] and has been implicated in aging sarcopenia [14] and inflammatory diseases [15, 16]. Conversely, increasing the antioxidant content through dietary vitamin/trace element supplements [14–17] or regular moderate-intensity training [18] alone or in combination [19] has improved both systemic oxidative stress or inflammation and muscle function. Significantly, supplementation concomitant with training was efficient only in subjects with preexisting antioxidant deficits [19].

Last, in contrast to healthy individuals, COPD patients experience baseline antioxidant (vitamin C, vitamin C/E ratio, zinc, and selenium) deficits [7, 20], which worsen after training [5]. Thus, training alone did not reduce the oxidative damage and inflammation in patients [5]. Given the role of antioxidants in muscle dysfunction and the muscles' lack of improvement with training, antioxidants constitute a candidate for the blunted muscle response to training in COPD. Currently, only one study has tested the combination of training and antioxidant supplementation in COPD patients and showed no additional effect of the combination versus training alone. However, the single supplemented antioxidant did not increase its cellular target (i.e., the GSH/GSSG ratio) [21] or improve the other antioxidant deficits observed in COPD patients [7, 20].

In order to assess the potential of combining exercise training and antioxidant supplementation to target antioxidant deficits in stable COPD patients [20], we therefore conducted a randomized double-blind controlled trial during a PR program. We specifically tested the effects of oral antioxidant supplementation with vitamins and trace elements (i.e., vitamins C and E, zinc, and selenium) versus placebo on muscle endurance (primary outcome) and muscle strength, oxidative stress, inflammation, and PR outcomes (secondary outcomes).

## 2. Material and Methods

### 2.1. Study Design, Randomization, and Ethics

In this 28-day monocentric trial, COPD patients were randomly allocated (1 : 1 ratio) with permuted blocks of four patients, ensuring an equal number of patients receiving antioxidant supplementation (PR antioxidant group) and placebo (PR placebo group) during a PR program. The program included exercise training and followed the recommendations for chronic respiratory patients [3]. All patients received a detailed information letter about the study before providing written informed consent. Randomization was carried out after consent was obtained and centralized by computer software at the medical information department of Montpellier University Hospital. Investigators, PR caregivers, the statistician, and COPD patients were blinded to the nature of supplementation until trial completion. The study was conducted in accordance with good clinical practice and the Declaration of Helsinki. It was approved by the ethics committee Montpellier Sud-Mediterranée IV (no. 2011-A00842-39). The trial was preregistered in https://www.clinicaltrials.gov (ClinicalTrials.gov identifier: NCT01942889).

### 2.2. Participants

Stable COPD patients (40 to 78 years old) referred to the *La Solane* Pulmonary Rehabilitation Center (5 Santé /Fontalvie Corporation, F-66340, Osséja, France) were recruited. COPD was defined by the occurrence of dyspnea, chronic cough or sputum production, and/or a history of exposure to risk factors for the disease and postbronchodilatator FEV_1_/FVC < 70%, evaluated by plethysmography (Body Box 5500, Medisoft, Belgium) according to the validated methodology [1]. The exclusion criteria were as follows: exacerbations within the last month, unstable disease incompatible with a PR program, antioxidant supplementation (vitamins, trace elements, etc.) or use of drugs such as allopurinol and N-acetylcysteine within the last month, and use of oral corticosteroids over the last six months. The BODE index was determined to assess global disease severity [7]. It was determined from the body mass index (B), the degree of airflow obstruction with FEV_1_ (O), dyspnea (D) as assessed by the Medical Research Council (MRC) scale, and exercise capacity (E) measured by the 6-minute walk test (6MWT).

### 2.3. Interventions

In line with the current guidelines for pulmonary rehabilitation, 24 sessions of endurance exercise (stationary cycling, walking) over 28 days were proposed [3]. Endurance training intensity was individualized for each patient and corresponded to the target heart rate at the ventilatory threshold [4], as assessed during a maximal cardiopulmonary exercise test. For each patient, the ventilatory threshold was assessed blindly by two investigators. This intensity was continuously monitored with a heart rate monitor, and the workload was progressively increased. Each session lasted 1 h and 30 min: 45 minutes of endurance training completed by 30 minutes of strength exercise (8-10 exercises, with sets of 10-15 repetitions) progressively increased using a perceived exertion scale (with a target of 5-6 on a 10-point scale) [4]. All sessions were supervised blindly by an experienced clinician. This exercise training was part of a multicomponent PR program. In order to promote long-term health behavior changes and long-term adherence, the PR also included dietary counseling, smoking cessation help, and educational interventions when needed [3].

Oral capsules of the antioxidant supplements or placebo were delivered to the patients daily by the nurses of the PR center. In accordance with the deficits observed previously in COPD patients [20], the antioxidant supplementation was associated with vitamin E (*α*-tocopherol: 30 mg/day), vitamin C (ascorbate: 180 mg/day), zinc gluconate (15 mg/day), and selenium (selenomethionine: 50 *μ*g/day). Although no vitamin E deficit was observed in the COPD patients, it was administered because of its synergistic effects with ascorbate [11]. Vitamin E was given as *α*-tocopherol because this is the form preferentially absorbed by humans, and it has more antioxidant effects than other tocopherols or tocotrienols [11]. The nutritional supplementation doses were in line with (selenium) or above (vitamins C and E and zinc) the Recommended Dietary Allowances and Adequate Intakes (Food and Nutrition Board) [22].

### 2.4. Outcomes

#### 2.4.1. Primary Outcome

The quadriceps endurance time (Qend, in seconds) may be more sensitive to interventions targeting the limb muscles, and the international guidelines therefore recommend its assessment in research studies [2]. The quadriceps endurance time was determined only for the dominant leg, as previously described by our group [8]. The patients performed knee extensions (6 movements per minute) with a workload to 30% of quadriceps maximal voluntary isometric contraction (QMVC) until exhaustion. Immediately after this test, they performed a QMVC to evaluate quadriceps fatigue, and a reduction in QMVC > 10% was necessary to validate the test, as previously published [8].

#### 2.4.2. Secondary Outcomes

Secondary outcomes included changes in (1) quadriceps maximal voluntary isometric contraction (QMVC) and (2) oxidative stress (plasma vitamin and trace elements, lipid peroxidation, and GSH/GSSG ratio), as previously described [20]. QMVC was compared with the reference values. The predicted QMVC in kg was given by the following regression equation:

(1) $$\begin{array}{c} \text{QMVC}=56.2-\left(0.306*\text{age in yrs}\right)+\left(0.686*\text{FFM in kg}\right)-\left(0.156*\text{height in cm}\right)-\left(3.42\text{ if female}\right). \end{array}$$

The residual standard deviation from the analysis was 8.58 kg, and patients with (observed-predicted QMVC)/8.58<−1.645 were considered weak [23].

In addition, we assessed validated parameters of inflammation, muscle mass, exercise capacity, and nutritional status, as previously described [20]. Briefly, the muscle mass was assessed by bioelectrical multifrequency segmental impedance (Biacorpus RX Spectral; Medical HealthCare GmbH, Karlsruhe, Germany), as previously described [20]. Muscle biopsies were performed in the *vastus lateralis* of the quadriceps before and after the interventions as previously described [4]. Muscle fiber type and mean cross-sectional area (CSA) were assessed after immunohistochemistry on frozen sections, using anti-MHCI monoclonal antibodies [4]. The nutritional status was assessed by the evaluations of the body mass index (BMI) and body composition by bioelectrical multifrequency impedance. Total serum protide, albumin, and prealbumin were determined on venous blood collected in dry tubes. Samples were immediately centrifuged and frozen (-80°C) until tested later. Total serum protide, albumin, and prealbumin were determined using an immune electrochemiluminescence assay on the Cobas 8000/e602® immunochemistry system (Roche Diagnostics, Meylan, France). Last, the inflammation was assessed by a dosage of highly sensitive C-reactive protein (hsCRP) on venous blood collected in dry tubes. Samples were immediately centrifuged and frozen (-80°C). Determination of CRP was run on the Cobas 8000/e502® analyzer (Roche Diagnostics, Meylan, France) using the immunoturbidimetric method [24].

Treatment compliance was recorded daily by the nurses. Adverse events, the number of patients experiencing acute exacerbations of COPD (defined as an acute worsening of respiratory symptoms that results in additional therapy) [1], and additional medications (indication, cumulative systemic corticosteroid dose, and treatment length) were recorded and examined as covariables. Last, physical training (training session attendance, intensity) and micronutrient intake are influenced by pulmonary rehabilitation interventions, and therefore, these confounding variables were also recorded before and during the study.

### 2.5. Sample Size and Statistical Analysis

Sample size was calculated based on the change in Qend in seconds [8]. We estimated that with a sample of 28 patients per group evaluated for the primary outcome, the study had at least 80% power to determine the superiority of the PR antioxidant group compared with the PR placebo group. For the intention-to-treat analysis, the following assumption was made: Qend change of 180 ± 120 s in the PR antioxidant group versus 90 ± 120 s in the PR placebo group. As we assumed a dropout rate of 10%, we included 32 stable COPD patients per group. Quantitative data are presented as mean ± standard deviation (SD), and qualitative data are described using proportions. To compare all the parameters between the PR placebo and PR antioxidant groups at baseline and to examine within-group relative variation, we used a Student *t*-test (or a Mann-Whitney for nonnormally distributed variables) for quantitative data and Pearson's chi-squared test for qualitative data. To assess the group effect on the relative variation, linear regressions were performed and covariable effects and their interactions were tested. If a confounder was identified, adjusted outcome analyses were performed by adding the confounder factor as a covariable of the test. For all analyses, the level of significance was set at *p* < 0.05. Statistical analyses were performed on an intention-to-treat basis using R version 3.3.1 and SAS version 9.3 (SAS Institute, Cary, NC).

## 3. Results

### 3.1. Study Flow Chart and Population

**Figure 1:**
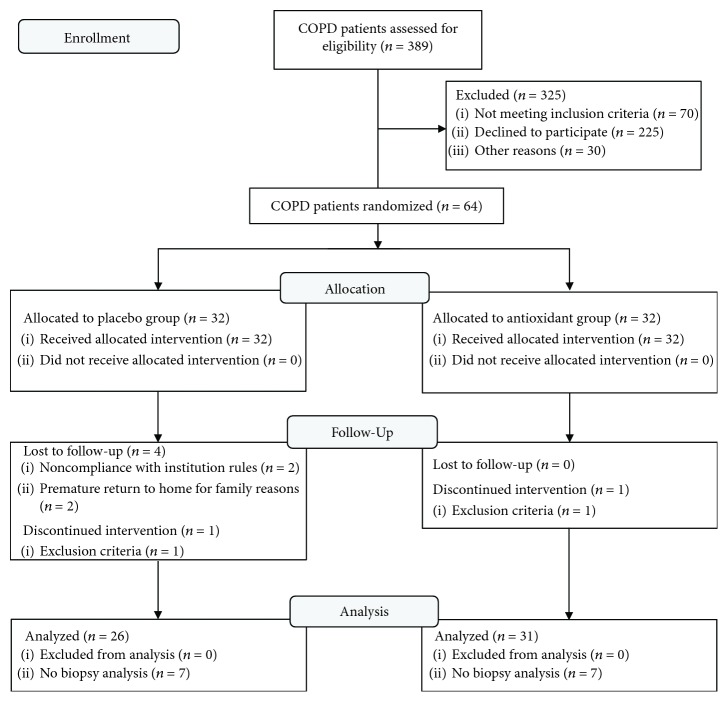
CONSORT diagram of the COPD patient recruitment.

Patients we enrolled between 3 June 2012 and 17 December 2014. As shown in the CONSORT flow diagram (Figure 1), 64 of the 389 screened COPD patients were randomized to receive placebo (PR placebo group, *n* = 32) or antioxidant supplementation (PR antioxidant, *n* = 32), 57 COPD patients completed the study and were included in the analysis, and 43 patients underwent muscle biopsy.

Participant characteristics and baseline measurements are shown in Tables 1 and 2.

When measured values were compared with predicted values, the COPD patients (49% males, FEV_1_ = 58.9 ± 20.0%pred) were exercise-intolerant (symptom-limited oxygen uptake (VO_2sl_) = 60.3 ± 16.4%pred and 6-minute walking distance (6MWD) = 80.0 ± 15.1%pred) and had muscle weakness (QMVC = 73.1 ± 19.7%pred). Based on the reference values, 81% had at least one nutritional antioxidant deficit, with 26%, 24%, and 67%, respectively, deficient in vitamin C, zinc, and selenium. As shown in Table 1, no significant differences were noted between the two groups for age, sex ratio, disease severity, body composition, or functional and skeletal muscle parameters. The GSH/GSSH ratio in the PR antioxidant group tended to be higher than that in the PR placebo group (454 ± 281 vs. 334 ± 241, respectively; *p* = 0.09, Table 2). The characteristics of the COPD patients who underwent muscle biopsy did not differ significantly from those of the whole group (Table 3).

As shown in Table 4, PR did not differ between groups in terms of training, treatment compliance, or micronutrient intake. However, despite a similar number of acute exacerbations, the PR placebo patients received more short-term systemic corticosteroid treatment than the PR antioxidant patients (31% vs. 6%, respectively; *p* < 0.02).

### 3.2. Effect of the PR Program

The significant improvements in quadriceps endurance and exercise capacity (symptom-limited workload: *W*_sl_ and 6MWD) in the PR placebo patients show that the 4-week moderate-intensity training program was efficient. In addition, the PR placebo group showed improved outcomes that were driven not only by training (Tables 5 and 6), as we observed improvements in vitamin C (9.4 ± 4.1 to 12.8 ± 3.3; *p* < 0.001) and GPx (45.4 ± 10.7 to 45.6 ± 8.6; *p* < 0.05).

### 3.3. Outcome Analysis

#### 3.3.1. Primary Outcome Analysis

The primary outcome Qend showed no additional increase in the PR antioxidant group (-6.6 ± 11.3%; *p* = 0.56). However, secondary outcome analysis revealed significant effects of the nutritional antioxidant supplementation (Tables 5 and 6).

#### 3.3.2. Secondary Outcome Analyses

**Figure 2:**
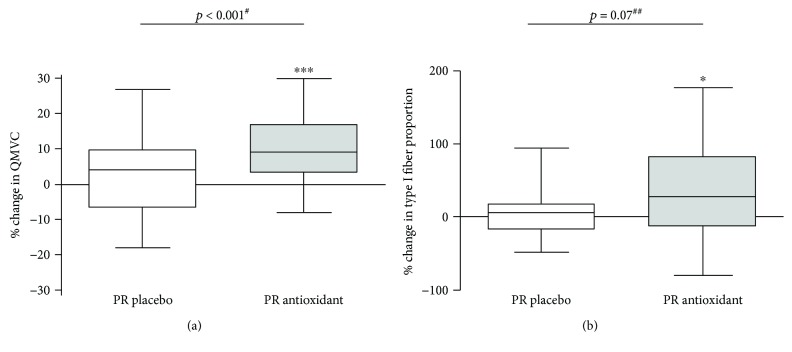
Box-and-whisker plots for relative change (%) in functional parameters: (a) quadriceps maximal voluntary contraction (QMVC) and (b) quadriceps type I oxidative fiber proportion. ^∗^*p* < 0.05, ^∗∗^*p* < 0.01, and ^∗∗∗^*p* < 0.001. ^#^Group effect adjusted on the corticosteroid treatment. ^##^Group effect adjusted on the baseline value and interaction term.

The corticosteroid-adjusted increases in QMVC were significant (Figure 2) in the PR antioxidant group (102 ± 38 to 111 ± 40 N·m^−1^; *p* < 0.001), whereas this change was not significant in the PR placebo group (114 ± 51 to 114 ± 51; *p* = 0.89). This resulted in a significant between-group difference in favor of antioxidant supplementation for QMVC (*p* < 0.001), after adjustment on the baseline difference and on the interaction between group and baseline values. The prevalence of muscle weakness decreased significantly (from 30.0 to 10.7%; *p* < 0.05) in the PR antioxidant group but not significantly in the PR placebo group (41.7 to 37.5%; *p* = 0.56), and this resulted in a significant between-group difference (*p* < 0.05).

**Figure 3:**
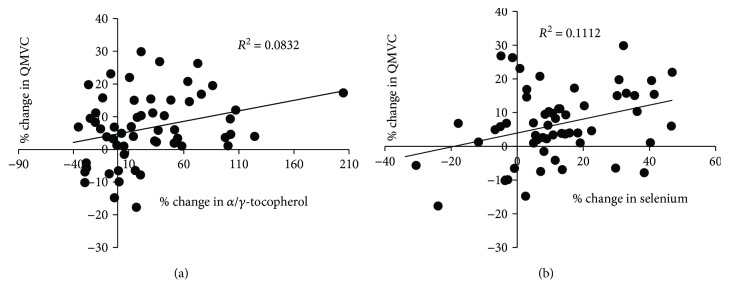
Linear regression analysis. The change (in %) in quadriceps maximal voluntary contraction (QMVC) was independently correlated with the change in the *α*-tocopherol/*γ*-tocopherol ratio (*r* = 0.39, *p* < 0.05) in all COPD patients (a), and the change (in %) in quadriceps maximal voluntary contraction (QMVC) was independently correlated with the change in selenium (*r* = −0.64, *p* < 0.001) in all COPD patients (b).

We observed increases in the *α*-tocopherol/*γ*-tocopherol ratio and the selenium level in the PR antioxidant group that were significantly greater than those in the PR placebo group (+51.1 ± 10.7%, *p* < 0.001, and +16.2 ± 4.5%, *p* < 0.01, respectively). We found significant and positive correlations between the change in QMVC and the change in the *α*-tocopherol/*γ*-tocopherol ratio (*r* = 0.39; *p* < 0.05) and between the change in QMVC and the change in selenium (*r* = 0.40; *p* < 0.05) (Figure 3). However, the change in markers of oxidative damage, such as lipid peroxidation, was not significant in the PR antioxidant group, and the magnitude of change did not differ from that in the PR placebo group (1.1 ± 13.1%, *p* = 0.93). There was a significant difference in the corticosteroid-adjusted change in the GSH/GSSG ratio. When the baseline values were taken into account, however, the change in the PR antioxidant group did not differ from that in the PR placebo group (-82 ± 61%; *p* = 0.19).

Although antioxidant supplementation did not significantly improve the muscle mass or fiber cross-sectional area, the proportion of the type I muscle fiber increased in the PR antioxidant group (34.4 ± 13.6% to 38.9 ± 16.0%; *p* < 0.05), whereas this change was not significant in the PR placebo group (41.4 ± 11.9% to 42.1 ± 11.6%; *p* = 0.89). This resulted in a trend for type I fiber proportion (*p* = 0.07), depicted in Figure 2.

Last, Tables 5 and 6 show the effect of antioxidant supplementation on serum total proteins versus placebo (+6.5 ± 2.4%; *p* < 0.01) but no significant effect of supplementation on other nutritional statuses or exercise capacity parameters.

## 4. Discussion

In this randomized double-blind, placebo-controlled trial, nutritional antioxidant supplementation (vitamins C and E, zinc, and selenium) failed to further improve the patients' quadriceps endurance (primary outcome). However, this study demonstrates additional improvements of three secondary outcomes (antioxidant deficits, QMVC, and serum total protein) and a trend toward increased type I fiber proportion with supplementation versus placebo during PR.

With the exception of the GSH/GSSG ratio, the two groups were comparable at baseline, particularly for the muscle function parameters. Specifically, the factors involved in COPD muscle dysfunction, like physical activity level, tobacco smoking, and vitamin/trace element intake [2], did not significantly differ between the randomized groups. Moreover, blinding was maintained throughout the study, which ensured comparability. Indeed, as shown in Table 4, the duration and compliance with treatment; the number, type, and intensity of the training sessions; and the micronutrient intake did not differ between groups. The tendency toward more patient dropouts in the placebo group (*p* = 0.052) was not related to the disease or the intervention (instead, to noncompliance with institutional rules and family reasons). Adverse events and acute exacerbations did not differ between groups. The only confounding factor was a higher prevalence of oral corticosteroid prescriptions in the placebo group (30.8% vs. 6.45%; *p* < 0.05). Given the positive correlation between short-term corticosteroid treatment and muscle strength [25], and its impact on oxidative stress and inflammatory parameters, a post hoc adjustment was performed for all secondary outcome muscle measures.

### 4.1. Impact of the Antioxidant Supplementation on the Serum Antioxidants

At baseline, more than 60% of the COPD patients had dietary intakes of vitamin C, vitamin E, and zinc below the recommendations, with the only exception being selenium. Moreover, we observed a baseline deficit in nutritional antioxidants in these patients, reproducing our previous findings [20]. After training, we found no increases in most of the enzymatic and nonenzymatic antioxidants in the PR placebo group, in agreement with previous studies in COPD [5]. However, vitamin C and GPx were increased in this group, consistent with the dietary counseling and education delivered during PR, as indicated by the high vitamin C and selenium intake in this nonsupplemented group (161 ± 71% and 171 ± 57% of the recommended dietary allowance, respectively). These increases in vitamin C and GPx may in part explain the lack of additional vitamin C improvement in the PR antioxidant group. Yet this latter group showed significant additional improvement in the *α*-tocopherol/*γ*-tocopherol ratio and plasma selenium, indicating that supplementation was efficient [26]. Zinc was also improved in the supplemented group, although the tendency for additional improvement versus placebo (*p* = 0.096) was not significant after adjustment on corticosteroid use (*p* = 0.390), which increases the plasma zinc level [24] and may thus have masked the effect of zinc supplementation in our study.

### 4.2. Impact of Antioxidant Supplementation on Muscle Function during PR

Exercise training was efficient because the increase in 6MWD (+44 ± 38 m; *p* < 0.001) was clinically significant in 62% (16/26) of the PR placebo patients [27]. Qend improved in both groups, and the adjusted difference between groups was not statistically significant (*p* = 0.56). Although we opted for the most sensitive muscle parameter to study pharmacological [28] and nonpharmacological interventions [4] targeting limb muscles [2], the sample size was based on the observed improvement in Qend after short-term antioxidant supplementation alone [8], which was the closest data available at the time for the design of our combined antioxidant and training study. However, given the large magnitude of Qend improvements in both the PR placebo and PR antioxidant groups (+37 ± 45% and +24 ± 43%, respectively), a ceiling effect of the training-induced increase in muscle function may have been reached, as recently reported for “add-on therapies” to training [29]. This effect was perhaps all the more notable as the PR placebo group was clearly not a training-only group but instead benefited from other interventions (such as dietary counseling and education). This ceiling effect was not observed for other secondary outcomes (except for the 6MWD), as shown by the nonsignificant changes observed in the PR placebo group. Conversely, the additional 11% improvement in QMVC in the PR antioxidant group was statistically significant and significantly correlated with changes in the *α*-tocopherol/*γ*-tocopherol ratio and selenium. This result is in agreement with previous studies showing that the greatest QMVC benefits have been observed in those with the largest antioxidant correction [19]. This further benefit of the “add-on” antioxidant supplementation is a striking result, as the improvements appear inconsistent in other PR studies on resistance training (alone or combined with endurance training) [30] and neuromuscular electrostimulation [31], and on balance, it caused no significant harm. Moreover, the QMVC improvement may be clinically significant because the proportion of COPD patients with muscle weakness decreased significantly from 30.0 to 10.7% in the PR antioxidant group, which was from a population of COPD patients attending a “real-life” PR program and showing a prevalence of muscle weakness similar to that of published cohorts [23]. Given the prognostic value of muscle weakness [2], we speculate that a similar decrease in its prevalence would be clinically relevant in large-scale studies with long-term outcome assessment. Last, given the heterogeneity of the antioxidant deficits in our COPD patients, a personalized antioxidant supplementation tailored to the precise antioxidant status of each individual [32] could also constitute a relevant perspective for further muscle improvement during training in COPD patients.

### 4.3. Muscle Pathways Induced by the Nutritional Supplementation during PR

Evidence of the greater benefit of nutritional intervention plus exercise training as opposed to combined training and placebo is scarce [2, 29], and our study is the first to show the greater improvement in muscle function with dietary antioxidants versus placebo in combination with exercise training. For example, the addition of targeted nutrition combining vitamin D/omega3 or leucine/vitamin D/omega3 with PR showed no additional benefit for exercise capacity, muscle mass, or muscle function [33]. Supplementation with antioxidant whey protein combined with exercise training showed no effect on the muscle [21]. In our study, the increases in *α*-tocopherol/*γ*-tocopherol and selenium with supplementation increased total serum protein, a hallmark of the protein catabolic state in COPD [34]. Given the similar protein intake in the two groups, this may have been a consequence of reduced protein catabolism and inflammation [35]. Although not documented by hsCRP, lipid peroxidation, and the GSH/GSSG ratio, the involvement of reduced inflammation or oxidative stress in the additional improvement in muscle strength cannot be ruled out, because not all the plasma and muscle markers of inflammation and oxidative stress were tested.

However, vitamin E and selenium do not only act as ROS scavengers and/or anti-inflammatory molecules [11, 12], but also modulate several pathways and cell functions like proliferation and apoptosis (PI3K/Akt/PPAR*γ*) [11, 36]. In this context, the additional increase in type I fiber proportion in the supplemented group merits discussion. Although the adjusted difference only tended toward significance compared with the PR placebo group (*p* = 0.07, power of the test: 37%), the difference might have been statistically significant had the full randomized groups been taken into account. Indeed, given that the lack of change in type I fiber proportion in our training-only group reproduced our past data [4] and the findings of other studies [5], completing the biopsy analyses in the PR placebo group would likely have confirmed the effect of supplementation on type I fiber proportion. The extensive literature on muscle diseases caused by deficiencies in vitamin E and selenium intake [37] is in line with this effect of *α*-tocopherol and plasma selenium on the muscle fiber type: white muscle disease, which is characterized by selective type I fiber destruction, is reversible with *α*-tocopherol and selenium supplementation in animals [38] and humans [39, 40]. Selenium supplementation during training induces muscle mitochondrial biogenesis in animals and humans [41]. Given the baseline selenium deficit and the increases in the *α*-tocopherol/*γ*-tocopherol ratio and selenium in the supplemented COPD patients, the oxidative fiber increase in this group could be imputed to supplementation.

## 5. Conclusions

In this double-blind randomized placebo-controlled trial, antioxidant supplementation associating vitamin E, vitamin C, zinc, and selenium was administered to COPD patients for 28 days in combination with pulmonary rehabilitation supplementation. This combination failed to improve the quadriceps endurance (primary outcome). Yet, it improved quadriceps muscle strength and serum total protein, and type I fiber proportion showed a trend toward increase. Thus, this is the first study to suggest that efficient antioxidant supplementation results in a clinically significant effect on a relevant outcome in PR (i.e., muscle strength), as well as other specific adaptations not explained by training alone. This study opens a field for more targeted antioxidant supplementation and “add-on” nutritional interventions in combination with exercise training during PR. Ancillary muscle analysis would provide greater understanding of the cell pathways involved in these skeletal muscle adaptations, such as a reduction in inflammation and/or oxidative stress or an adaptation of the muscle oxidative phenotype.

## Figures and Tables

**Table 1:** Baseline clinical, functional, and muscle characteristics of the randomized study population.

	PR placebo group (*n* = 26)	PR antioxidant group (*n* = 31)	*p* value
Age (years)	61.1 ± 8.7	62.4 ± 6.5	0.55
Sex ratio (W/M)	13/13	16/15	0.90
BMI (kg/m^2^)	25.3 ± 4.7	25.0 ± 4.2	0.78
Fat-free mass index (kg/m^2^)	17.3 ± 2.4	17.4 ± 2.3	0.85
Muscle mass index (kg/m^2^)	7.9 ± 1.6	8.0 ± 1.5	0.74
FEV_1_ (%pred)^∗^	62 ± 27	57 ± 17	0.41
FEV_1_/FVC ratio^∗^	43 ± 14	41 ± 10	0.99
BODE score	1.9 ± 1.7	2.2 ± 1.9	0.60
Breathlessness (MRC score)	1.2 ± 0.9	1.3 ± 1.2	0.81
Tobacco consumption (pack years)	40 ± 18	45 ± 26	0.63
Physical activity level score	6.0 ± 6.7	5.7 ± 4.9	0.85
6MWD (m)	508 ± 106	504 ± 112	0.96
6MWD (%pred)	79 ± 17	81 ± 16	0.64
*W*_sl_ (watt)	70 ± 30	60 ± 25	0.17
*W*_sl_ (%pred)	52 ± 19	51 ± 18	0.84
VO_2sl_ (ml/min/kg)	16.6 ± 4.0 (*n* = 18)	14.1 ± 3.4 (*n* = 7)	0.16
VO_2sl_ (%pred)	66 ± 16 (*n* = 18)	56 ± 15 (*n* = 7)	0.16
VO_2_ at VT (ml/min/kg)	10.1 ± 2.7	9.5 ± 2.77	0.42
QMVC (N·m)	114 ± 51	114 ± 51	0.52
QMVC (%pred)	72.8 ± 21.9	74.0 ± 21.6	0.39
Qend (s)	355 ± 158	343 ± 117	0.75
Type I muscle fiber %	41 ± 12	34 ± 14	0.09
Type I muscle fiber CSA (*μ*m^2^)	5497 ± 1689	4810 ± 1824	0.22
Non-type I muscle fiber CSA (*μ*m^2^)	4307 ± 1740	4118 ± 1408	0.84
All muscle fiber CSA (*μ*m^2^)	4839 ± 1589	4369 ± 1549	0.34

^∗^Postbronchodilatation. Results are expressed as mean ± SD. Definition of abbreviations: COPD: chronic obstructive pulmonary disease; PR: pulmonary rehabilitation; W/M: women/men; BMI: body mass index (kg/m^2^); FEV_1_ (%pred): forced expiratory volume in 1 second; BODE index: body mass index, airway obstruction, dyspnea, exercise capacity index (6MWD); MRC: Medical Research Council; 6MWD (m): six-minute walking distance; *W*_sl_: symptom-limited power output; VO_2sl_: symptom-limited oxygen uptake; VT: ventilatory threshold; QMVC (N·m^−1^): quadriceps maximal voluntary contraction expressed in Newtons; Qend (s): quadriceps endurance time expressed in seconds; CSA: cross-sectional area expressed in *μ*m^2^.

**Table 2:** Baseline serum oxidative stress and inflammation characteristics of the randomized study population.

	PR placebo group (*n* = 26)	PR antioxidant group (*n* = 31)	*p* value
Vitamin C (*μ*g/ml) (W: 8.6-18.8; M: 6.2-15.2)	9.5 ± 4.1	10.8 ± 4.7	0.29
*α*-Tocopherol (mg/l) (8.0-15.0)	14.1 ± 3.0	15.7 ± 4.5	0.12
*γ*-Tocopherol (mg/l)	1.03 ± 0.33	1.18 ± 0.55	0.75
*α*-Tocopherol/*γ*-tocopherol ratio	14.6 ± 4.0	15.2 ± 6.0	0.99
Vitamin C/*α*-tocopherol (0.59-1.19)	0.71 ± 0.34	0.71 ± 0.33	0.97
Selenium (*μ*g/ml) (94-130)	84.2 ± 14.0	87.6 ± 14.6	0.37
Copper (mg/ml) (W: 0.8-1.55; M: 0.7-1.40)	1.13 ± 0.26	1.11 ± 0.21	0.76
Zinc (mg/ml) (0.70-1.20)	0.82 ± 0.12	0.79 ± 0.13	0.30
Copper/zinc ratio (1.14-1.29)	1.41 ± 0.42	1.45 ± 0.37	0.45
GSH/GSSG ratio (111-747)	334 ± 241	454 ± 281	0.09
GPx (UI/gHb) (30-55)	45.4 ± 10.7	49.3 ± 11.7	0.20
SOD (UI/gHb) (785-1570)	1308 ± 351	1286 ± 250	0.84
Lipid peroxidation (*μ*mol/l) (<432)	652 ± 283	625 ± 309	0.74
LDLox (UI/l) (28-70)	59 ± 21	61 ± 18	0.44
hsCRP (mg/l)	5.5 ± 6.3	6.8 ± 10.9	0.57
Total serum protides (g/l)	68.8 ± 5.8	66.6 ± 5.5	0.13
Serum albumin (g/l)	47.4 ± 4.7	46.2 ± 8.3	0.84
Serum prealbumin (g/l)	0.278 ± 0.069	0.293 ± 0.069	0.47

Results are expressed as mean ± SD. In the first column, we specify the lower and upper limits of reference values obtained in a large healthy subject cohort, as previously described [10]. Definition of abbreviations: PR: pulmonary rehabilitation; M: male; W: women; GSH: reduced glutathione; GSSG: oxidized glutathione; SOD: superoxide dismutase; GPx: peroxidase glutathione; LDL: oxidized low-density lipoprotein.

**Table 3:** Characteristics of the COPD patients who underwent muscle biopsy.

	PR placebo group (*n* = 26)	Biopsy PR placebo group (*n* = 19)	*p* value	PR antioxidant group (*n* = 31)	Biopsy PR antioxidant group (*n* = 24)	*p* value
Age (years)	61 ± 9			62 ± 6		
Sex ratio (W/M)	13/14	11/8	1.00	17/15	13/11	1.00
BMI	25.4 ± 4.7	24.6 ± 4.3	0.54	24.8 ± 4.3	25.8 ± 4.1	0.39
FEV_1_ (%pred)	62 ± 27	61 ± 32	0.67	55 ± 18	51 ± 18	0.91
6MWD (%pred)	78 ± 17	78.1 ± 17	0.95	81 ± 14	84 ± 13	0.58
VO_2sl_ (%pred)	64 ± 16	64 ± 17	0.90	56 ± 16	55 ± 16	0.84
QMVC (%pred)	90 ± 24	99 ± 21	0.18	87 ± 14	87 ± 15	0.97
*α*/*γ*-Tocopherol	15.7 ± 4.1	14.6 ± 4.0	0.39	15.2 ± 6.0	15.9 ± 6.1	0.69
Selenium	85.4 ± 14.2	84.9 ± 14.2	0.91	88.3 ± 14.3	89.8 ± 13.2	0.70
Qend (s)	357 ± 155	367 ± 174	0.82	345 ± 116	359 ± 122	0.66

Delta QMVC (%)	+3 ± 11%	+1 ± 11%	0.60	+9 ± 9%	+10 ± 9%	0.79
Delta *α*/*γ*-tocopherol (%)	-4 ± 24%	-9 ± 24%	0.49	+53 ± 47%	+54 ± 51%	0.93
Delta selenium (%)	+2 ± 17%	0 ± 17%	0.61	+20 ± 14%	+20 ± 14%	0.92

Results are expressed in mean ± SD. Definition of abbreviations: W/M: women/men; BMI: body mass index (kg/m^2^); FEV_1_ (%pred): forced expiratory volume in 1 second; 6MWD (m): six-minute walking distance; VO_2sl_: symptom-limited oxygen uptake; QMVC (N·m^−1^): quadriceps maximal voluntary contraction expressed in Newtons; Qend (s): quadriceps endurance time expressed in seconds; CSA: cross-sectional area expressed in *μ*m^2^.

**Table 4:** Confounding factors during the study of the randomized study population.

	PR placebo group (*n* = 26)	PR antioxidant group (*n* = 31)	*p* value
Systemic corticosteroid treatment	8/18 (31%)	2/29 (6%)	0.02
Duration of corticosteroid treatment (days)	4 ± 1.4	5 ± 1.41	0.33

Antioxidant/placebo supplementation			
Duration (days)	26.4 ± 1.8	26.7 ± 0.7	0.59
Capsule consumption (*n*)	112 ± 3	112 ± 2	0.91
Compliance (%)	99 ± 2	99 ± 1	0.37

Exercise training intensity			
VO_2sl_ at VT/pred. VO_2sl_ (%)	42 ± 12	42 ± 16	0.44
Number of exercise training sessions			
(i) stationary cycling	8 ± 2	8 ± 2	0.83
(ii) walking	8 ± 3	8 ± 3	0.39
(iii) strength building	10 ± 3	10 ± 4	0.70
(iv) adapted physical activity	10 ± 4	8 ± 3	0.11

Daily micronutrient intake			
Vitamin C (mg/d)	179 ± 80	201 ± 81	0.46
Vitamin C (%RDA)	161 ± 71%	183 ± 74%	
Vitamin E (mg/d)	5.7 ± 1.6	5.3 ± 1.7	0.56
Vitamin E (%RDA)	47 ± 13%	44 ± 14%	
Zinc (mg/d)	10.3 ± 3.1	10.0 ± 2.8	0.79
Zinc (%RDA)	97 ± 32%	87 ± 24%	
Copper (mg/d)	1.4 ± 0.5	1.5 ± 0.5	0.60
Copper (%RDA)	86 ± 29%	88 ± 31%	
Selenium (*μ*g/d)	100.6 ± 33.8	98.3 ± 29.8	0.85
Selenium (%RDA)	171 ± 57%	162 ± 49%	

Exacerbations during interventions			
Duration (days)	5 ± 3	4 ± 3	0.49

Results are expressed as mean ± SD. Definition of abbreviations: PR: pulmonary rehabilitation; VO_2sl_: symptom-limited oxygen uptake; VT: ventilatory threshold; RDA: Recommended Dietary Allowances and Adequate Intakes.

**Table 5:** Outcome assessment: clinical, functional, and muscle parameters.

	PR placebo group (*n* = 26)			PR antioxidant group (*n* = 31)			Mean difference of relative change	Adjusted mean difference of relative change^#^
	Pre	Post	Relative change (%)	Pre	Post	Relative change (%)		
Qend (s)	355 ± 158	457 ± 177	37.4±45.1^∗∗∗^	343 ± 117	430 ± 175	27.4±36.0^∗∗∗^	-10.1 ± 10.7	-6.6 ± 11.3
QMVC (N·m^−1^)	114 ± 51	114 ± 51	-0.4 ± 15.8	102 ± 38	111 ± 40	9.4±8.9^∗∗∗^	9.7±3.3^∗∗^	11.1±3.5^∗∗^
Muscle mass index (kg/m^2^)	7.9 ± 1.6	7.9 ± 1.6	0.0 ± 4.5	8.0 ± 1.5	7.9 ± 1.6	-0.4 ± 3.4	-0.4 ± 1.1	-0.4 ± 1.1
Muscle fiber cross-sectional area (*μ*m^2^)	4839 ± 1589	4674 ± 1216	2.3 ± 30.3	4369 ± 1549	4836 ± 1928	18.1 ± 49.5	15.9 ± 13.2	17.5 ± 14.4
Type I muscle fiber proportion (%)	41.4 ± 11.9	42.1 ± 11.6	5.5 ± 30.1	34.4 ± 13.6	38.9 ± 16.0	32.8 ± 72.0^∗^	27.3 ± 18.1	31.8 ± 19.6
6MWD (m)	508 ± 106	553 ± 109	9.2±8.6^∗∗∗^	504 ± 112	537 ± 106	7.9±9.4^∗∗∗^	-1.3 ± 2.4	-1.4 ± 2.6
*W*_sl_ (W)	70 ± 30	77 ± 31	2.8 ± 12.7	60 ± 25	66 ± 28	1.3 ± 9.5	-1.4 ± 3.0	-0.7 ± 3.2
VO_2sl_ (ml/min/kg)	16.6 ± 4.0 (*n* = 18)	17.1 ± 5.1 (*n* = 18)	2.1 ± 13.9	14.1 ± 3.4 (*n* = 7)	15.6 ± 3.0 (*n* = 7)	11.4 ± 11.8^∗^	9.4 ± 6.0	7.7 ± 6.1
VO_2_ at VT (ml/min/kg)	10.4 ± 2.9	11.1 ± 4.3	21.9 ± 80.8	9.5 ± 2.8	11.6 ± 2.8	90.1±322.0^∗∗^	68.2 ± 69.4	58.0 ± 73.1
BMI (kg/m^2^)	25.3 ± 4.7	25.1 ± 4.7	-0.9 ± 2.9	24.9 ± 4.3	24.6 ± 4.0	-1.0 ± 2.2^∗^	-0.1 ± 0.7	-0.4 ± 0.7
Fat-free mass index (kg/m^2^)	17.3 ± 2.4	17.3 ± 2.5	0.1 ± 2.7	17.4 ± 2.3	17.4 ± 2.3	0.5 ± 3.3	0.5 ± 0.8	0.6 ± 0.9

^∗^ *p* < 0.05, ^∗∗^*p* < 0.01, and ^∗∗∗^*p* < 0.001. ^#^Mean adjusted value on the corticosteroid treatment ± SEM. Definition of abbreviations: *W*_sl_ (% pred): symptom-limited power output; Vo_2sl_ (% pred): symptom-limited oxygen uptake; VT: ventilatory threshold.

**Table 6:** Outcome assessment: inflammation and oxidative stress markers.

	PR placebo group (*n* = 26)			PR antioxidant group (*n* = 31)			Mean difference of relative change	Adjusted mean difference of relative change^#^
	Pre	Post	Relative change (%)	Pre	Post	Relative change (%)		
Vitamin C (*μ*g/ml)	9.5 ± 4.1	12.8 ± 3.3	72.1±113.9^∗∗^	10.8 ± 4.7	15.1 ± 2.8	86.9±133.0^∗∗^	14.9 ± 33.2	5.1 ± 35.0
*α*-Tocopherol (mg/l)	14.1 ± 3.0	14.4 ± 4.1	2.5 ± 23.2	15.7 ± 4.5	17.3 ± 4.1	13.9±25.4^∗∗^	11.3 ± 6.5	11.6 ± 6.9
*γ*-Tocopherol (mg/l)	1.03 ± 0.33	1.12 ± 0.49	10.4 ± 34.5	1.18 ± 0.55	0.88 ± 0.37	-19.5±27.3^∗∗∗^	-29.9±8.2^∗∗∗^	-24.5±8.4^∗∗^
*α*-Tocopherol/*γ*-tocopherol ratio	14.6 ± 4.0	13.6 ± 3.8	-2.9 ± 23.1	15.2 ± 6.0	22.1 ± 7.6	53.2±47.6^∗∗∗^	56.1±10.2^∗∗∗^	51.1±10.7^∗∗∗^
Vitamin C/*α*-tocopherol	0.71 ± 0.34	0.95 ± 0.33	75.6±128.9^∗∗^	0.71 ± 0.33	0.91 ± 0.24	66.9±112.4^∗∗^	-8.6 ± 32.0	-15.3 ± 33.9
Selenium (*μ*g/ml)	84.2 ± 14.0	86.0 ± 12.2	3.8 ± 16.2	87.6 ± 14.6	104.1 ± 12.4	20.5±15.8^∗∗∗^	16.7±4.2^∗∗∗^	16.2±4.5^∗∗∗^
Copper (mg/ml)	1.13 ± 0.26	1.14 ± 0.36	1.1 ± 23.0	1.11 ± 0.21	1.11 ± 0.24	1.3 ± 18.3	0.3 ± 5.5	-1.6 ± 5.8
Zinc (mg/ml)	0.82 ± 0.12	0.84 ± 0.14	3.7 ± 16.3	0.79 ± 0.13	0.86 ± 0.13	9.7±12.8^∗∗∗^	6.0 ± 3.9	3.4 ± 4.0
Copper/zinc ratio	1.41 ± 0.42	1.37 ± 0.46	-0.9 ± 28.1	1.45 ± 0.37	1.32 ± 0.33	-6.6 ± 19.9	-5.7 ± 6.4	-4.5 ± 6.8
GSH/GSSG ratio	334 ± 241	433 ± 266	182.5 ± 303.4^∗^	454 ± 281	385 ± 312	11.8 ± 114.1	-170.7±62.2^∗∗^	-155.6 ± 64.8^∗^
GPx (UI/gHb)	45.4 ± 10.7	45.6 ± 8.6	6.3 ± 13.6^∗^	49.3 ± 11.7	53.0 ± 12.0	8.7±14.8^∗∗^	2.4 ± 3.9	4.4 ± 4.2
SOD (UI/gHb)	1308 ± 351	1388 ± 367	17.5 ± 46.2	1286 ± 250	1313 ± 306	7.3 ± 28.4	-10.1 ± 15.0	4.2 ± 13.7
Lipid peroxidation (*μ*mol/l)	652 ± 283	708 ± 357	15.2 ± 46.0	625 ± 309	640 ± 340	13.3 ± 46.7	-1.9 ± 12.3	1.1 ± 13.1
LDLox (UI/l)	59 ± 21	57 ± 17	2.5 ± 27.1	61 ± 18	62 ± 16	4.0 ± 20.1	1.4 ± 6.3	-0.4 ± 6.6
hsCRP (mg/l)	5.5 ± 6.3	7.3 ± 7.9	374.3 ± 1430.5	6.8 ± 10.9	4.6 ± 5.1	137.1 ± 496.3	-237.2 ± 314.6	-68.0 ± 339.1
Total serum protides (g/l)	68.8 ± 5.8	67.7 ± 4.7	-1.6 ± 8.1	66.5 ± 5.5	69.1 ± 5.0	3.7 ± 8.1^∗^	5.3 ± 2.3^∗^	6.5±2.4^∗∗^
Serum albumin (g/l)	47.4 ± 4.7	46.7 ± 6.0	3.5 ± 17.5	46.2 ± 8.3	47.7 ± 4.3	0.9 ± 12.6	-2.6 ± 4.7	-4.8 ± 5.1
Serum prealbumin (g/l)	0.278 ± 0.069	0.261 ± 0.053	4.2 ± 31.1	0.293 ± 0.069	0.270 ± 0.048	-2.2 ± 19.0	-6.4 ± 7.7	-11.7 ± 8.1

^∗^ *p* < 0.05, ^∗∗^*p* < 0.01, and ^∗∗∗^*p* < 0.001. ^#^Mean adjusted value on the corticosteroid treatment ± SEM. Definition of abbreviations: hsCRP: highly sensitive C-reactive protein; GSH: reduced glutathione; GSSG: oxidized glutathione; SOD: superoxide dismutase; GPx: peroxidase glutathione; LDL: oxidized low-density lipoprotein.

## Data Availability

The data used to support the findings of this study are available from the corresponding author upon request.
